# Mantle cell lymphoma of the gastrointestinal tract presenting with multiple intussusceptions – case report and review of literature

**DOI:** 10.1186/1477-7819-7-60

**Published:** 2009-07-31

**Authors:** Venkata KN Kella, Radu Constantine, Nalini S Parikh, Mary Reed, John M Cosgrove, Stephen M Abo, Saundra King

**Affiliations:** 1 Department of Surgery and Oncology, Bronx-Lebanon Hospital Center, Bronx, New York, USA; 2 Department of Surgery, Pathology and Medical Oncology, Saint Michaels Medical Center, Newark, New Jersey, USA

## Abstract

**Background:**

Mantle cell lymphoma (MCL) is an aggressive type of B-cell non-Hodgkin's lymphoma that originates from small to medium sized lymphocytes located in the mantle zone of the lymph node. Extra nodal involvement is present in the majority of cases, with a peculiar tendency to invade the gastro-intestinal tract in the form of multiple lymphomatous polyposis. MCL can be accurately diagnosed with the use of the highly specific marker Cyclin D1. Few cases of mantle cell lymphoma presenting with intussuception have been reported. Here we present a rare case of multiple intussusceptions caused by mantle cell lymphoma and review the literature of this disease.

**Case presentation:**

A 68-year-old male presented with pain, tenderness in the right lower abdomen, associated with nausea and non-bilious vomiting. CT scan of abdomen revealed ileo-colic intussusception. Laparoscopy confirmed multiple intussusceptions involving ileo-colic and ileo-ileal segments of gastrointestinal tract. A laparoscopically assisted right hemicolectomy and extended ileal resection was performed. Postoperative recovery was uneventful. The histology and immuno-histochemistry of the excised small and large bowel revealed mantle cell lymphoma with multiple lymphomatous polyposis and positivity to Cyclin D1 marker. The patient was successfully treated with Rituximab-CHOP chemotherapy and remains in complete remission at one-year follow-up.

**Conclusion:**

This is a rare case of intestinal lymphomatous polyposis due to mantle cell lymphoma presenting with multiple small bowel intussusceptions. Our case highlights laparoscopic-assisted bowel resection as a potential and feasible option in the multi-disciplinary treatment of mantle cell lymphoma.

## Background

Approximately 6% of lymphomas are classified as mantle cell lymphomas (MCL) [1,2]. MCL generally occurs in adults with a median age of 60 and a male predominance. Advanced disease with involvement of regional lymph nodes, liver, spleen, or peripheral blood is common at presentation. More than 50% of patients with MCL have bone marrow involvement at the time of diagnosis. The primary presentation of extra nodal disease occurs in one quarter of patients and frequently involves Waldeyer's ring and the gastrointestinal tract. Multiple lymphomatous polyposis (MLP) is one of the most common primary gastrointestinal presentations of MCL and accounts for approximately about 9% of primary gastrointestinal lymphomas [3].

MLP most commonly occurs in the ascending colon and the small bowel, particularly in the ileum and ileocecal region. Occasionally, however, numerous polyps are present throughout the entire gastrointestinal tract. Polyps may be sessile, polypoid or both. They range in size from 0.1 to 4–5 cm and present with ulceration.

Intussusception occurs when a proximal segment of bowel (intussusceptum) telescopes into the lumen of an adjacent distal segment (intussuscipiens) and can occur anywhere within the gastrointestinal tract. Although fairly common in children, adult intussusception is relatively rare representing only 1% of patients with bowel obstructions [4,5]. We present a case of multiple lymphomaotous polyposis due to mantle cell lymphoma presenting with multiple intussusceptions.

## Case presentation

A 68-year-old previously healthy male presented with four days of constant pain in the right lower abdomen, associated with nausea and vomiting. There was no history of fever or weight loss. Physical examination revealed normal vital signs, a soft distended abdomen with hyperactive bowel sounds, and a palpable tender mass in the right lower quadrant. Digital rectal examination revealed hemorrhoids and guaiac positive stool. Laboratory evaluation was notable for low hematocrit (31%) and albumin (2.6 g/dL) levels. A plain abdominal radiograph showed a nonspecific gas pattern in the bowel with fecal loading of the descending and sigmoid colon.

**Figure 1:**
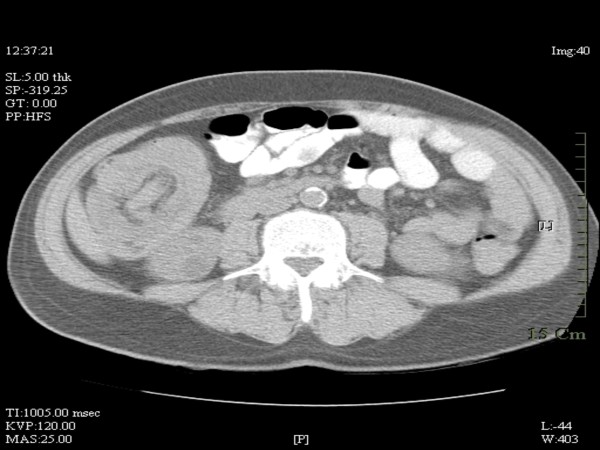
**CT scan of abdomen showing ileo-colic intussusception**.

**Figure 2:**
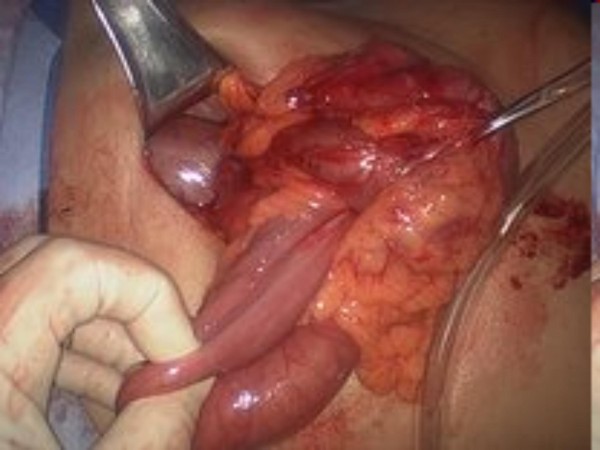
**Intraoperative pictures showing multiple (three) intussusceptions (part 1)**.

**Figure 3:**
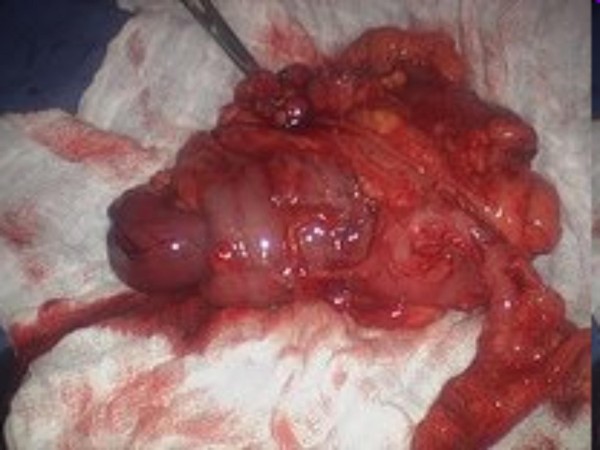
**Intraoperative pictures showing multiple (three) intussusceptions (part 2)**.

**Figure 4:**
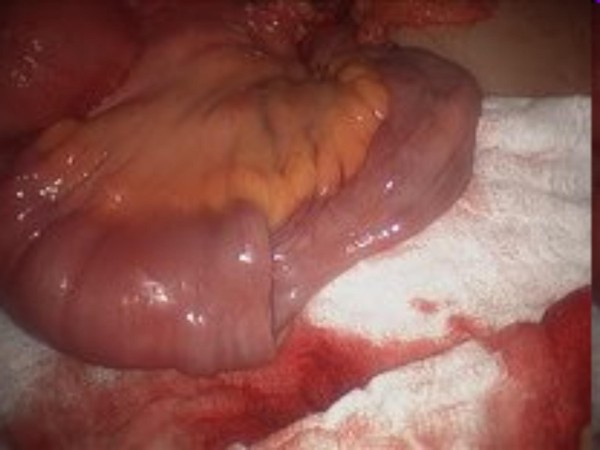
**Intraoperative pictures showing multiple (three) intussusceptions (part 3)**.

A CT-scan of the abdomen with contrast showed ileo-colic intussusception (Fig 1). At laparoscopy, ileocecal intussusception and two more ileo-ileal intussusceptions were found along with multiple tumors involving the entire length of jejunum, ileum and ascending colon (Figures 2, 3, 4).

The patient underwent a laparoscopically-assisted right hemicolectomy, with extended ileal resection and a stapled ileo-colic anastomosis. The postoperative period was uneventful and the patient was discharged on the fourth postoperative day.

**Figure 5:**
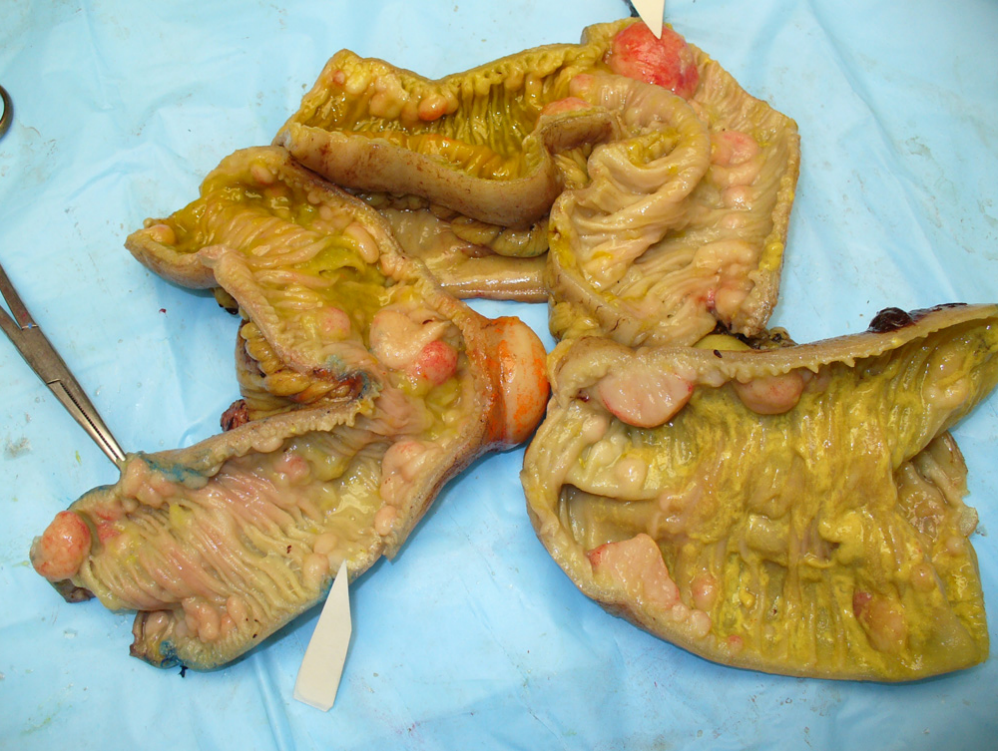
**Excised specimen showing numerous intraluminal and serosal lymphomatous polyposis**.

**Figure 6:**
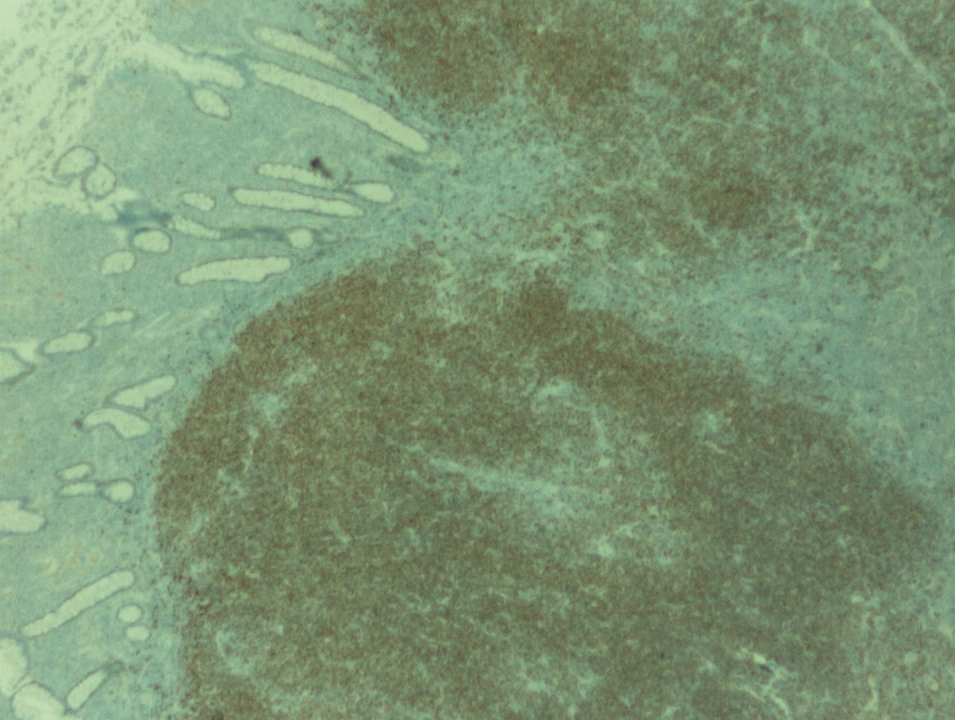
**Immunohistochemistry of the polypoid lesion revealing strong positivity with Cyclin D1**.

**Figure 7:**
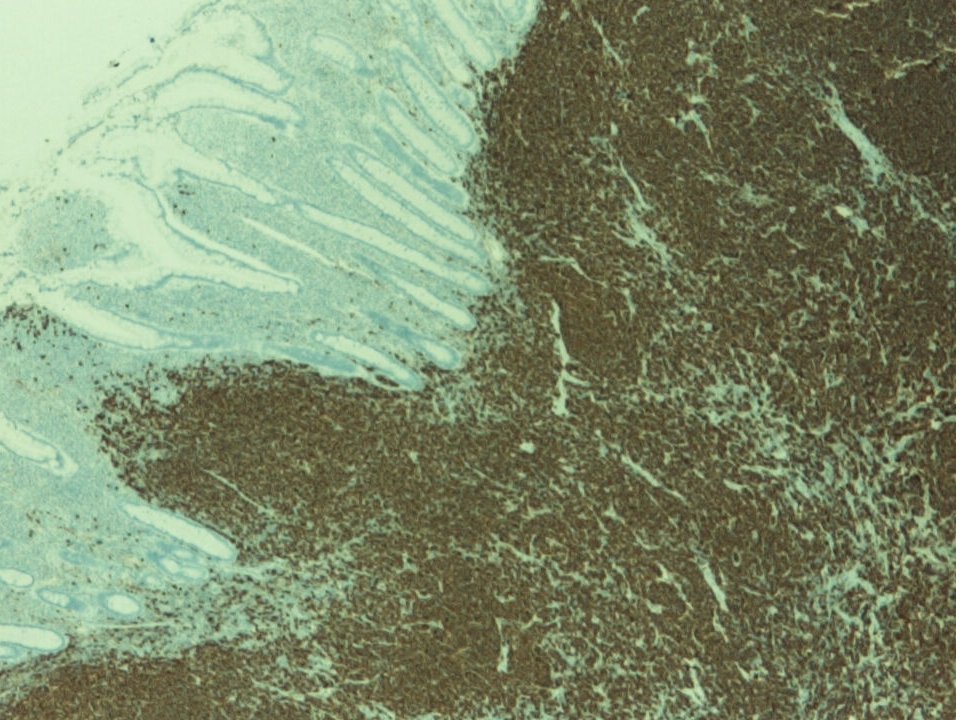
**Immunohistochemical stain with CD 20 showing strong positivity**.

**Figure 8:**
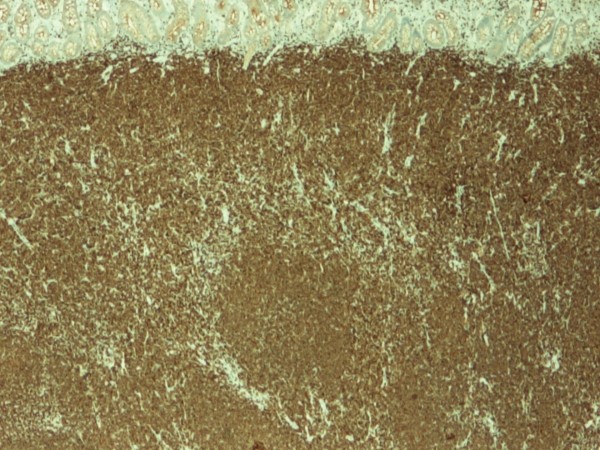
**Immuno histochemical stain with CD5 showing strong reactivity**.

**Figure 9:**
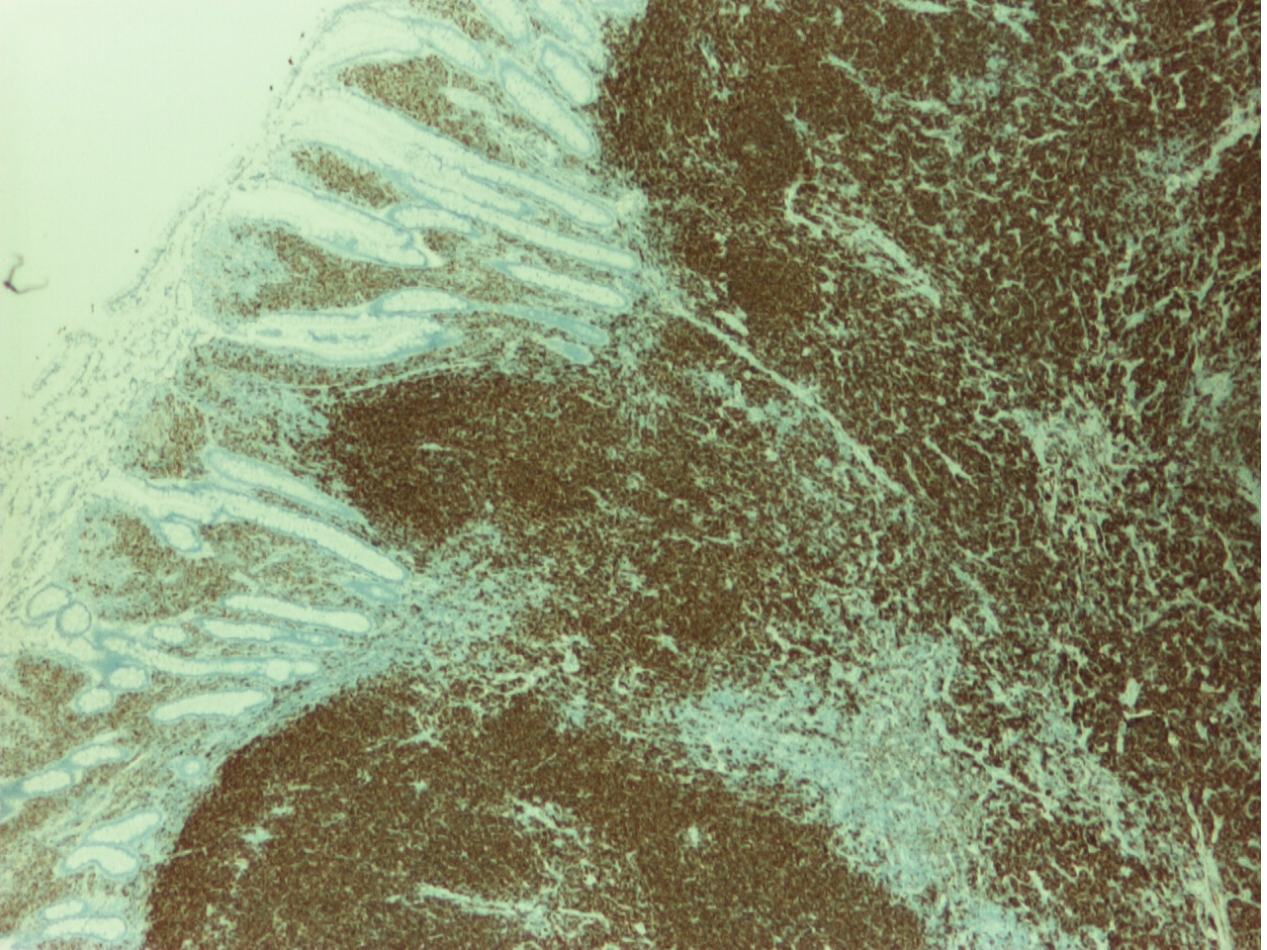
**Immuno histochemical stain with CD79a showing strong positivity**.

**Figure 10:**
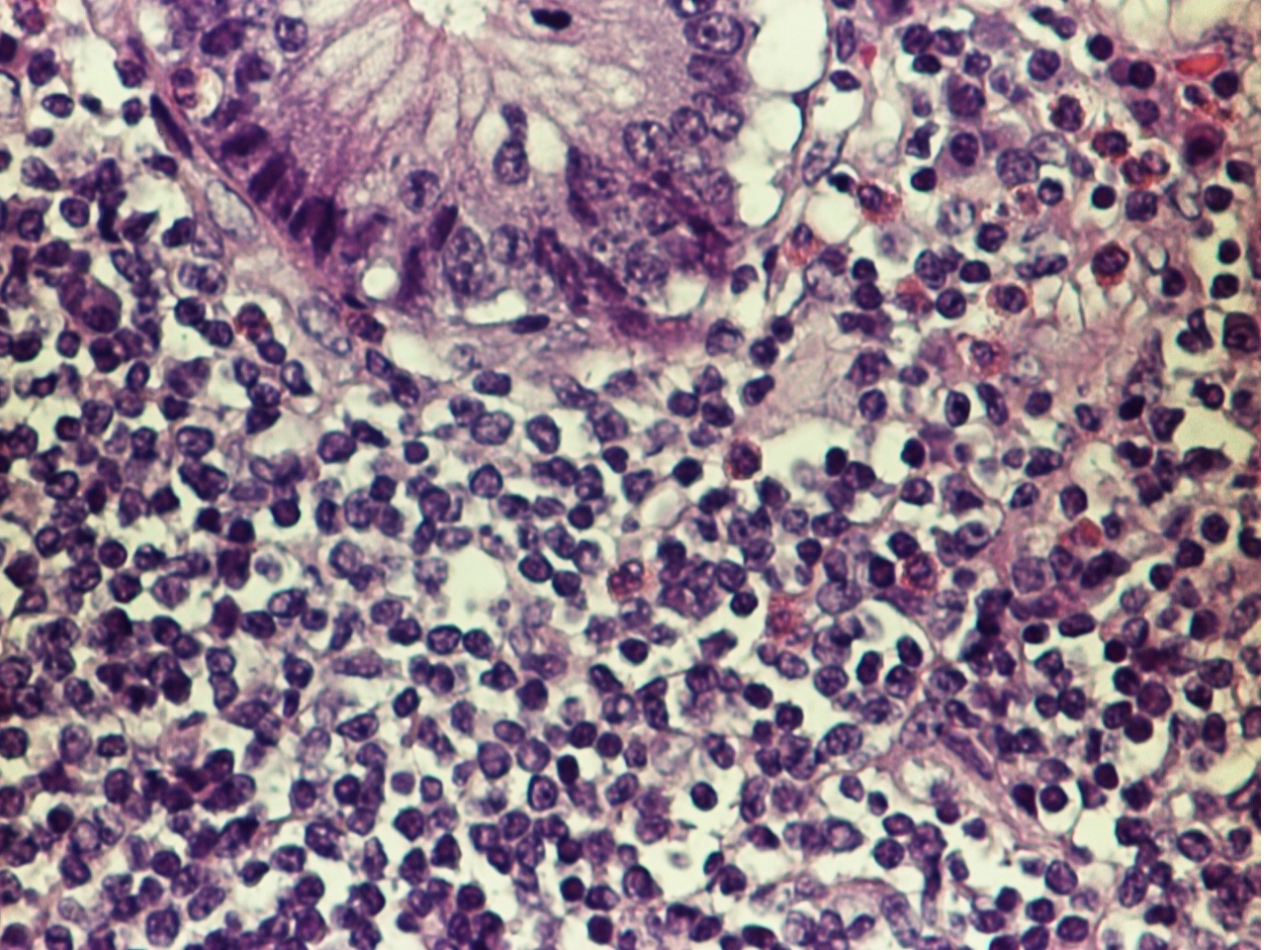
**Cytological appearance of mantle cell lymphoma High power field**. The tumor is composed of small to medium sized lymphocytes.

The pathology confirmed multiple lesions of about one inch diameter, involving the small bowel, cecum, and asceding colon (Fig 5). Histology revealed a malignant B-cell lymphoma. Immuno histochemistry and immunophenotypic analyses were positive for Cyclin D1 (Fig 6), CD20 (Fig 7), CD5 (Fig 8) and CD 79a (Fig 9), but negative for BCL6, CD23 and CD10, thus confirming the diagnosis of mantle cell lymphoma (Fig 10).

Upon hospital discharge, the patient underwent staging investigations with negative bone marrow involvement. He received 6 out of 8 planned cycles of chemotherapy with rituximab-CHOP (cyclophosphamide, doxorubicin, vincristine, prednisone) with moderate to severe toxicity in form of fatigue, febrile neutropenia despite growth factors and dose reduction, and failure to thrive. Restaging with PET CT confirmed complete response with no residual disease. At one year follow-up, he remains in remission with a good performance status.

## Discussion

The term "multiple lymphomatous polyposis" was first presented by Cornes in 1961 to describe numerous polypoid lesions throughout the entire GI tract consisting of mucosal involvement by malignant lymphoma [3]. In 1980, Blackshaw classified MLP as B-cell centrocytic non-Hodgkin's lymphoma according to the Kiel classification [6,7] According to the Working Formulation; MLP is classified as a diffuse, small-cleaved cell malignant lymphoma [8]. Isaacson et al. [9] and Triozzi et al. [10] have suggested that MLP is the digestive counterpart of mantle zone lymphoma that arises in lymph nodes. Following further immunohistochemical and cytogenetic study, MLP has been confrmed to be a mantle cell lymphoma involving the gastrointestinal tract [10].

MLP can present with symptoms such as abdominal pain, diarrhea, bleeding, and less frequently, protein-losing enteropathy, intestinal malabsorption, or chylous ascites. Rarely, MLP presents as an acute abdomen due to perforation or intestinal obstruction. MLP polyps usually occur in the ileocecal region and in one third of cases present as a mass [11,12]. Upper gastrointestinal endoscopy, enteroscopy and colonoscopy are important tools in diagnosing MLP to assess the locations of the polyps and obtain tissue biopsies. Differentiating lymphomatous polyposis from adenomatous or hamartomatous polyposis by endoscopic or radiological evaluation alone is impossible and tissue diagnosis is required. Additionally, not all lymphomatous polyposis of the gastrointestinal tract result from MCL. Michopulos et al, showed that only 12 out of 35 cases of lymphomatous polyposis were MCL [13]. Follicular lymphoma and MALT lymphoma can also present with MLP.

Definitive diagnosis of MLP requires histological examination of the specimen with histomorphologic and immunophenotypic analysis. In our case, histological examination of the ileum, colon, mesenteric mass, and lymph nodes showed malignant B-cell non-Hodgkin's lymphoma. The histo morphology and the immunophenotypic analysis were consistent with a mantle cell lymphoma – positive for Cyclin D1, CD20, CD79a and CD5; negative for BCL16, CD23 and CD10. These immunomarkers are essential in distinguishing mantle cell from other types of lymphoma. See table 1[14].

**Table 1:** Phenotypic markers and chromosomal translocations in common B-cell lymphomas.

	S Ig	CD5	CD10	CD20	Other	BCL2	Cyclin D1	K aryotype	Oncogene Function
B-CLL/SLL	Weak	+	-	Weak	CD23+ FMC7-		-	Deltion Trisomy	
Follicular	++	-	+	+	+	+	-	t (14;18)	BCL2 Antiapoptosis
Mantle cell	++	+	-	+	FMC7+	+	++	t (11;14)	Cyclin D1 Cell cycle regulator
Marginal Zone	+	-	-	+	CD11c+/-		-	No consistant anomaly	
Nodal Marginal zone	+	-	-	+	CD11 c+/-		-	t (11;18)	AP12/MALT1 Antiapoptosis
MALT large cell	+	-	-	+		+/-	-	t (1;14) t(3;14); t(3;v)	BCL10 Antiapoptosis
									BCL 6/BCL2 Transcription Factor
Burkitt	+	-	+/-	+	TdT-	-	-	t (8;14) t(2,8) t (8;22)	CMYC Transcription factor

Additionally, cytogenetic analysis of MCL shows rearrangement of the bcl-1 locus on chromosome11 due to t (11:14) (q13:q32) translocation, accompanied by cyclin D1 antigen overexpression [15].

Surgery is the mainstay of therapy for intussusception in adult patients. Increasingly, laparoscopy is replacing open operations as the preferred approach. Diagnostic laparoscopy may assist in the diagnosis of intussusception in cases where diagnosis is suspected but not confirmed by preoperative workup [16]. If the diagnosis is confirmed, then appropriate surgical therapy and resection can be performed depending on the comfort level of the surgeon. Laparoscopy may aid in planning the incision if a laparoscopic-assisted or even laparotomy incision is required.

MCLs usually respond poorly to conventional therapeutic regimens and are associated with short median survival. Current combinations of monoclonal antibodies and multi-agent chemotherapy have achieved significant improvement in MCL response rates. Overall response rates range from 80% to 95% and complete response rates of 30% to 50% are frequently being achieved [17]. The R-CHOP regimen was chosen due to the patient's poor performance status at the time of diagnosis. Our patient only received 6 out of 8 planned cycles of R-CHOP due to toxicity. Other chemotherapy regimens such as R-Hyper-CVAD (Rituxan with hyperfractionated cyclophosphamide, vincristine, doxorubicin, and dexamethasone alternating with high-dose methotrexate and cytarabine) have shown good results in uncontrolled trials. However, it is a more aggressive regimen associated with increased toxicity. Despite the improved high response rate, current overall survival rates remain poor because of the early relapse. Median survival with standard treatment for MCL patients remains between 3 and 4 years [18]. Intensive immuno-chemotherapy both with and without stem cell support has been successfully used to prolong the progression-free survival to 5 or more years [19,20]. These approaches along with other innovative strategies utilizing bortezembin [21], temsirolimus [22] or radioimmuno conjugates for the relapsed or refractory setting remain under active investigation.

To our knowledge, this is the first reported case of MCL presenting with multiple intussusceptions of gastrointestinal tract, separately involving the ileo colic and ileo-ileal segments. Our case highlights laparoscopic-assisted bowel resection as a potential and feasible option in the multi-disciplinary treatment of mantle cell lymphoma, when intussusception from MLP occurs.

## Abbreviations

MCL: Mantle cell lymphoma; MLP: Multiple lymphomatous polyposis;

## Consent

Written informed consent was obtained from the patient for publication of this case report and accompanying images. A copy of the written consent is available for review by the Editor-in-Chief of this journal

## Competing interests

The authors declare that they have no competing interests.

## Authors' contributions

VK, wrote the manuscript and involved in the patient care, RC performed the surgery, NP reviewed the histology and contributed to manuscript, SA involved with chemotherapy and contributed to revision of manuscript, MR, JC Critical review of manuscript. All authors read and approved the manuscript.
